# A Seven-Year Retrospective Study on the Surveillance of Hepatitis B in Laos

**DOI:** 10.1155/2018/9462475

**Published:** 2018-04-01

**Authors:** Phimpha Paboriboune, Thomas Vial, François Chassagne, Philavanh Sitbounlang, Sengaloun Soundala, Stéphane Bertani, Davone Sengmanothong, Francois-Xavier Babin, Nicolas Steenkeste, Paul Dény, Pascal Pineau, Eric Deharo

**Affiliations:** ^1^Centre d'Infectiologie Lao-Christophe Mérieux, Vientiane, Laos; ^2^IRD, UPS, UMR 152 PHARMADEV, Université de Toulouse, Toulouse, France; ^3^Fondation Mérieux, Lyon, France; ^4^Hôpitaux Universitaires Paris Seine Saint Denis, Université Paris 13, Sorbonne Paris Cité, Paris, France; ^5^INSERM U1052, CNRS UMR 5286, Cancer Research Center of Lyon, Lyon, France; ^6^Institut Pasteur, Organisation Nucléaire et Oncogenèse, Paris, France

## Abstract

**Objective:**

Lao PDR is one of the most highly endemic countries for hepatitis B in Asia and the second country for liver cancer incidence. Therefore, the follow-up of infected individuals through predictive serological markers is of utmost importance to monitor the progression of the pathology and take the decision on treatment.

**Methods:**

A retrospective-descriptive cohort study was conducted on 3,857 HBV-infected patients. Information about infection status (viral load, VL), liver function (aminotransferases), and treatments was recorded.

**Results:**

M/F sex ratio was 1.77 for a median age of 37. Patients under 37 displayed higher VL than older ones and men had higher VL than women. Initial VL ranged from <50 IU/mL to 2.5 10^13^ IU/mL. Median aminotransferase values were 45.5 U/L for ALAT and 44 U/L for ASAT, ranging from <8 to >2,000 U/L. Men had higher aminotransferase than women. Globally 20% of patients received treatment (mainly immunostimulant and reverse-transcriptase inhibitors); 11% had high levels of VL and liver enzymes, but only 2% of them were treated.

**Conclusion:**

Public health decisions should be taken urgently to rationalise vaccination and provide fair access to early diagnosis and treatment; otherwise the burden of HBV-associated diseases will be overwhelming for Laos in the near future.

## 1. Background

Hepatitis B (HBV) is a viral infection encountered all around the world, especially in Southeast Asia where the prevalence of persistent HBV infection is particularly high [1]. Despite efforts made to decrease the level of endemicity in the population through vaccination campaigns [2], HBV infection rates are still elevated in Laos [3]. This situation raises concerns, as chronic HBV infection leads to severe hepatic complications, such as cirrhosis and hepatocellular carcinoma (HCC). According to the Asia-Pacific HCC Trials Group, over two-thirds of people dying annually of HCC are from Asia [4]; and according to GLOBOCAN, Laos has one of the most elevated rates of liver cancer worldwide, after Mongolia which ranks first [5]. Clinically, HCC patients have a very poor prognosis with an appalling 5-year mortality rate due to HCC [6]. Consequently, early detection, serial monitoring, and appropriate treatment of HBV-infected patients are keys in order to control the burden of HCC.

With the objective of estimating the efficacy of HBV surveillance in Laos, we undertook a survey at* Centre d'Infectiologie Lao-Christophe Mérieux* (CILM) in the Lao PDR capital Vientiane. Under the auspices of the Ministry of Health, CILM is devoted to the surveillance of infectious diseases among the Lao population, notably viral hepatitis, human immunodeficiency virus (HIV), and tuberculosis.

In the present study, we examined the course of HBV infection in 3,857 patients attending CILM between January 2010 and November 2016, who were previously found to be positive for the surface antigen of HBV (HBsAg). Several parameters, such as the gender, age, geographic place of living, treatment allocation, HBV DNA viral load (VL), and liver damage (as measured by aspartate (ASAT) and alanine (ALAT) aminotransferases serum levels) were analyzed.

The results presented herein are intended to help policy makers and stakeholders to apply cost-effective preventive and treatment measures against HBV and its severe health consequences in Lao PDR.

## 2. Methods

### 2.1. Data Collection

The present study was conducted retrospectively within a cohort assembled with the data of HBV-infected Lao patients attending CILM between January 2010 and November 2016.

All individuals were previously diagnosed as HBsAg-positive in local health care facilities and advised to go for HBV VL monitoring at CILM. It must be pointed out that HBeAg was not tested among these patients because this kind of test is not available in the health centres in Laos.

Sociodemographic data, health care structure location, date of HBV diagnosis, treatment history, aminotransferase (ASAT/ALAT), and viral load were registered into a database set up with Filemaker Pro Version 11. All data were exported to Microsoft Excel software version 14.4.7 to check any incongruity.

### 2.2. Statistics

Data were analyzed with Minitab software version 17.3.1. Comparisons between groups (numerical data) or proportions (categorical data) were performed using Student's *t*-test, Chi-squared test, ANOVA, or nonparametric test, as appropriate. A two-sided *p* value lower than 0.05 was considered to be statistically significant.

## 3. Results

### 3.1. Cohort

**Figure 1:**
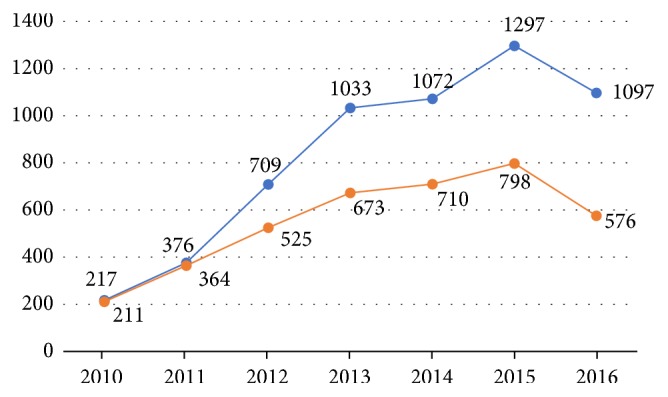
Number of patients followed (orange line) and number of samplings received and performed at CILM (blue line) from January 2010 to November 2016.

**Figure 2:**
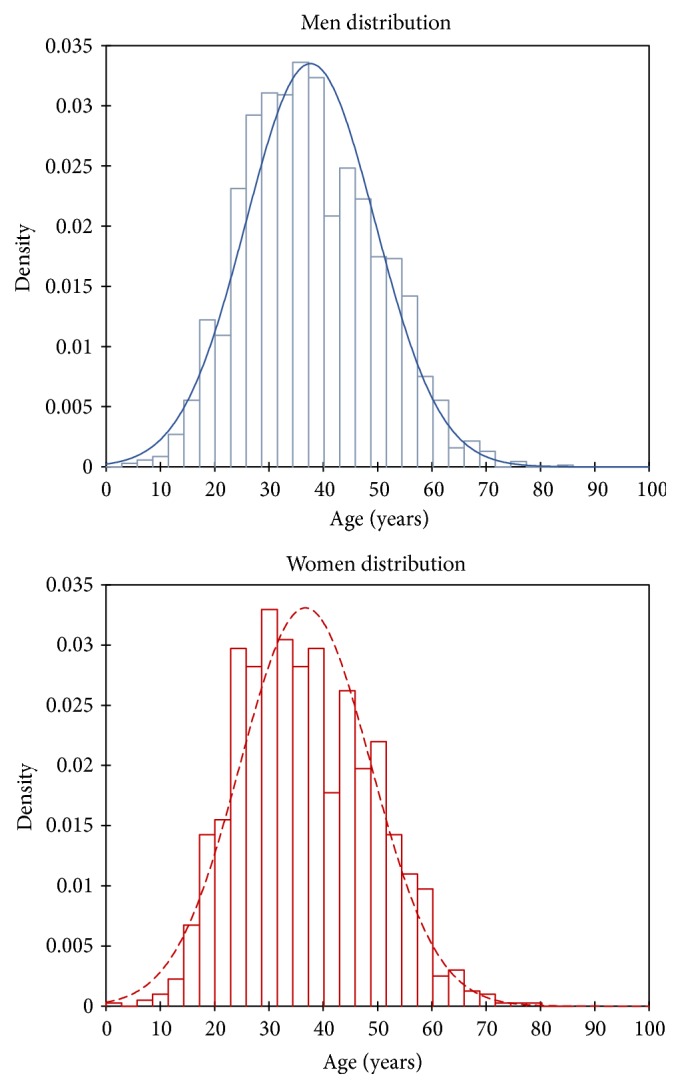
Age-based distribution of patients followed at CILM according to their gender (blue: male; red: female).

The laboratory received a total of 5,801 blood samples, from 3,857 patients between January 2010 and November 2016 (Figure 1). The age of patients ranged from 1 to 85 years, and median ages were 37 and 36 years for men and women, respectively (Figure 2). The cohort displayed a M/F ratio of 1.77.

About 70% of the individuals included in the survey came from public health care centres and 30% of them from the private health system. A vast majority (91%) of individuals were from Vientiane capital and only 9% of them came from provincial health care centres.

The number of follow-up blood samples varied from one to seventeen per patient, with interval between sampling ranging from one day to several months.

### 3.2. Laboratory Tests

#### 3.2.1. HBV DNA Quantification

All 3,857 patients had at least one VL measurement, 23.3% (*n* = 898) had two measurements, 11.3% (*n* = 436) had three, 6% (*n* = 230) had four, 3.6% (*n* = 139) had five, and 2.3% (*n* = 88) had six or more assays. The mean duration to second determination of the VL was one year.

**Figure 3:**
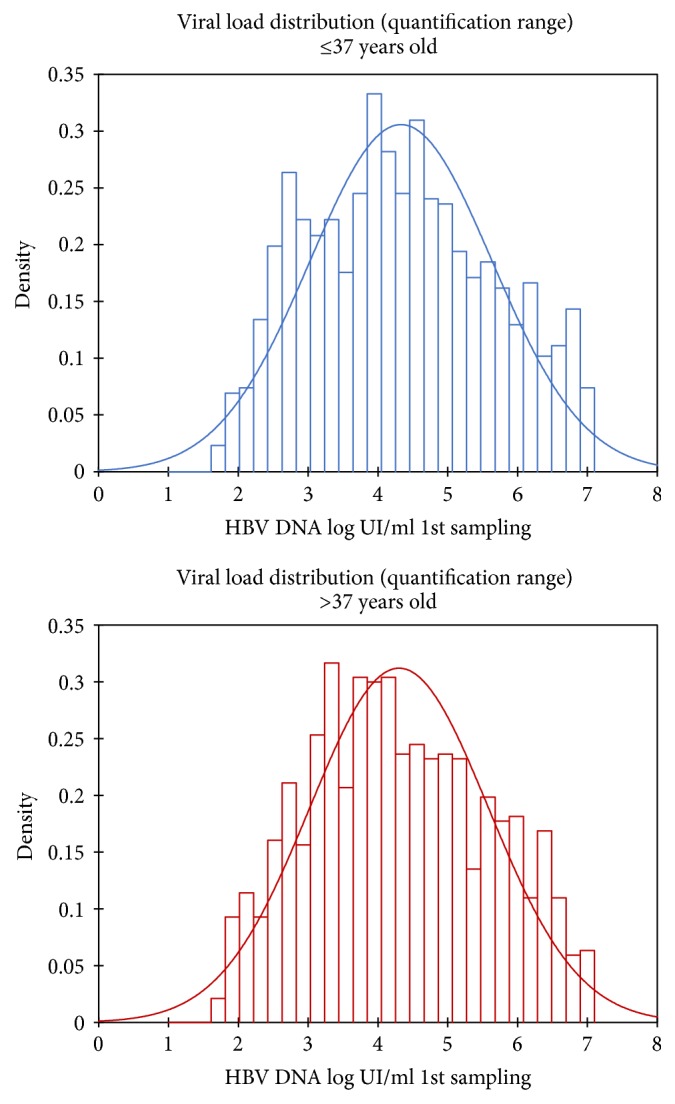
Distribution of the viral load at first sampling, according to age, in people from the “Detectable and Quantifiable” category.

**Figure 4:**
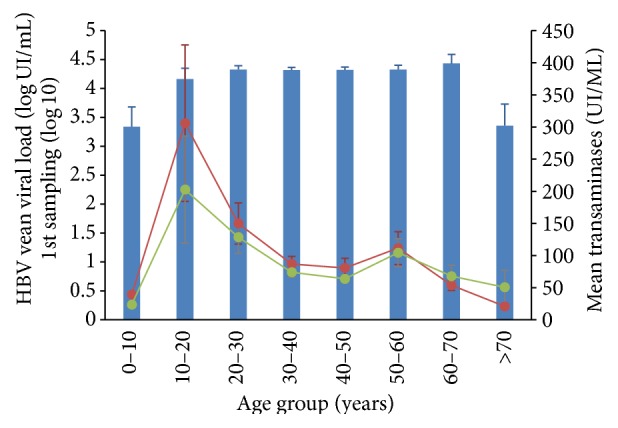
Mean viral load (blue bars) and aminotransferases (ALAT in red, ASAT in green) according to groups of age (ten years) at first sampling in the* DQ* category (only patients with VL* and* aminotransferases, excluding patients without aminotransferases).

VL determination at the first follow-up sampling ranged from less than 50 to 2.5 10^13^ IU/mL. Individuals were stratified in three different classes, according to their VL at first follow-up sampling:

(i) The HBV DNA Undetectable (UD) class had 672 patients (17%) for whom the VL was <50 IU/mL. M/F ratio for this class was 1.22, and 47% of the patients were under age 37. A large majority of them, that is, 83% (*n* = 562), were untreated. Thirteen percent of the individuals of this class (*n* = 90) had a second follow-up sampling, which had a median VL value of 14,270 IU/mL.

(ii) The HBV DNA Detectable and Quantifiable (DQ) class, with a VL ranging between 50 and 10^7^ IU/mL, represented the majority of the patients with 58% (*n* = 2,229) (Figures 3 and 4). The median value for this class was 42,234 IU/mL. M/F ratio for this class was 1.77, and 48% of the patients were under the age of 37. As observed for UD class, a very large majority of them, that is, 84% (*n* = 1,872), remained untreated. Twenty-four percent of this class (*n* = 551) had had a second follow-up sample, which had a median VL value of 40,035 IU/mL.

(iii) The HBV DNA Nonprecisely Quantifiable (NQ) class consisted of the patients for whom the VL was above 10^7^. The NQ class represented 25% of the initial cohort of patients (*n* = 956). There were significantly more men than women in this class (*p* < 0.0001, 69% for men versus 39% for female). Sixty-nine percent of the NQ individuals (*n* = 659) were under age 37. Despite the extremely high VL, only 24% (*n* = 228) of them received treatment. Thirty percent (*n* = 286) had a second follow-up sample: 5.2% had an undetectable VL and 94.8% had a VL ranging from 18,000 to 40,000 IU/mL. NQ patients were significantly younger than those from UD and DQ classes (*p* < 0.0001), whereas there was no statistical difference based on age between UD and DQ individuals (*p* = 0.079).

#### 3.2.2. Aminotransferase Levels

**Figure 5:**
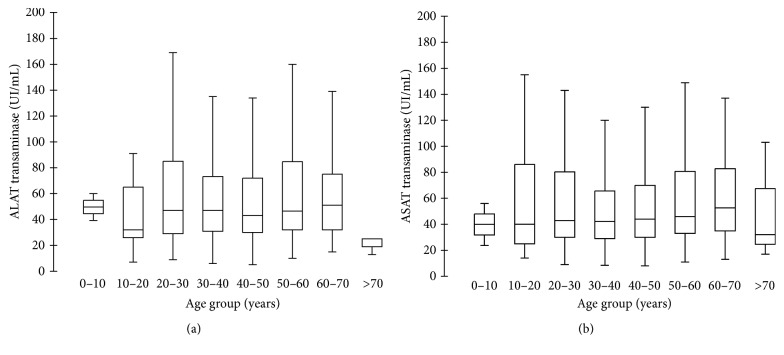
ASAT (a) and ALAT (b) concentrations according to 10-year classes of age of patients.

For aminotransferase, 40 U/L was considered as the upper limit of laboratory reference (ULN) [7]. ALAT values ranged from 5 to 3,071 U/L, with a median value of 45.5 U/L. ASAT values ranged from 8 to 2,057 U/L, with a median value of 44 U/L (Figures 4 and 5). Overall, median aminotransferase values were significantly higher in men than in women (*p* < 0.0001, ALAT for men, 812 U/L, versus ALAT for female, 457 U/L).

Patients were age-stratified in 10-year subsets, and aminotransferase levels were compared accordingly. ALAT and ASAT at first follow-up sampling were independent of age (*p* > 0.05). We then stratified aminotransferase values according to VL classes, as both VL and aminotransferases were monitored in 1,269 patients.

(i) In the UD class, 185 patients (out of 193) had a single aminotransferase determination. Both ALAT and ASAT median values were 42 IU/mL, with ranges from 7 to 1,025 U/L for ALAT and 7 to 1,855 U/L for ASAT. Eight patients had a second follow-up aminotransferase determination, with median values of 46 and 49.5 U/L for ALAT and ASAT, respectively. Both ALAT and ASAT aminotransferases had a lowest value around 30 U/L and highest one around 100 U/L.

(ii) In the DQ class, 744 patients benefited from aminotransferase determination. The median values were 42 U/L for ALAT and 40 U/L for ASAT, with lowest values around 7 U/L and highest ones over 2,000 U/L for both aminotransferases. 70 patients had a second follow-up aminotransferase determination, with median values of 57.7 and 51.25 U/L for ALAT and ASAT, respectively. Both ALAT and ASAT aminotransferases had a lowest value around 7 U/L and a highest one around 200 U/L.

(iii) In the NQ class, 340 NQ patients benefited from aminotransferase determination. The median values were 54 U/L for ALAT and 53.5 U/L for ASAT, with lowest values around 10 U/L and highest ones over 1,000 IU/mL for both aminotransferases. Forty-four patients had a second follow-up ALAT determination (median value: 42 U/L) and 43 patients had a second follow-up ASAT determination (median value: 38 U/L). The lowest values were around 6 U/L for both ASAT and ALAT and highest ones 296 and 170 U/L, respectively. NQ patients were younger and displayed higher ALAT and ASAT levels than UD and DQ patients (*p* < 0.0001). While considering aminotransferases levels above 40 U/L as abnormal, there were significantly more men with elevated aminotransferase level over 40 U/L than women (*p* < 0.0001). On the contrary, there were more women than men in the patient population with aminotransferases level lower than 40 U/L (*p* < 0.0001). Furthermore, there was no difference in terms of VL between patients displaying aminotransferases levels lower and higher than the 40 U/L threshold level (*p* = 0.052).

### 3.3. Treatments

Physicians followed and adapted the recommendations of the American Association for the Study of Liver Diseases [8]. In absence of a national health insurance system, treatments prescribed were thus reflective of the availability of drugs in Laos, as well as the capacity of the patients to pay for them.

#### 3.3.1. Evolution of Therapeutic Schemes

**Figure 6:**
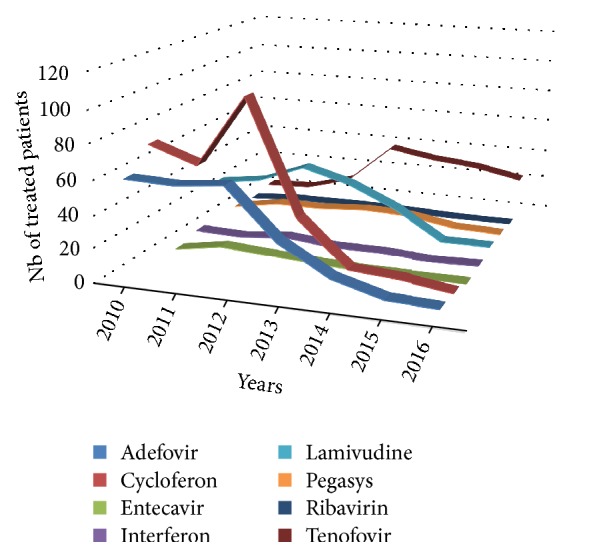
Evolution of the number of patients treated and medicines administered to patients received at CILM between January 2010 and November 2016.

**Figure 7:**
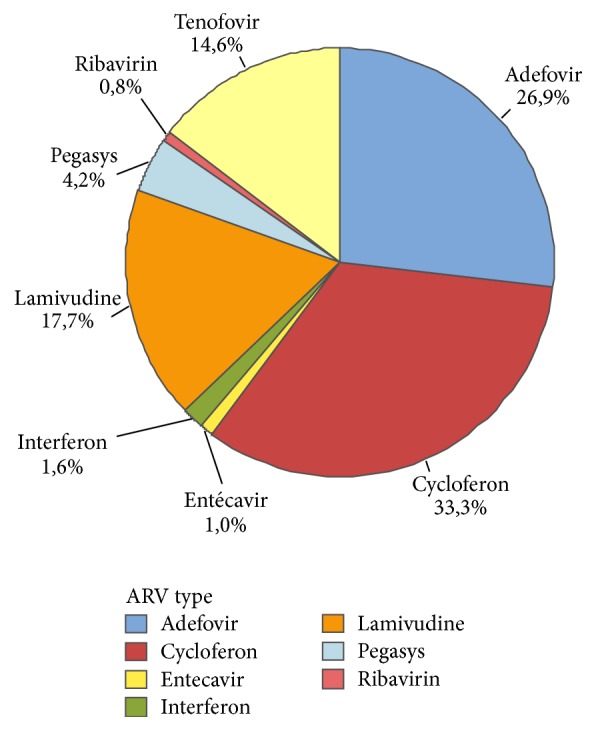
Distribution of drugs (%) used in all therapeutic schemes (single, dual, and triple).

**Figure 8:**
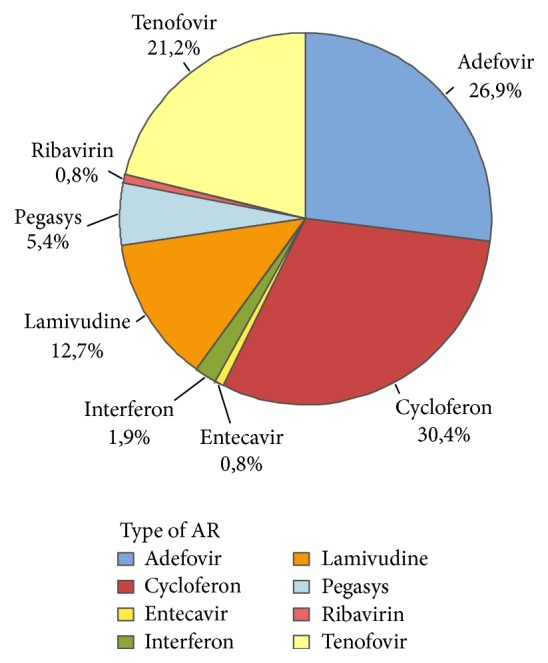
Distribution of drugs (%) used in monotherapy.

The evolution of antiviral drug prescription in Laos between 2010 and 2016 is detailed in Figure 6. Overall, only 18% (693 out of 3857) of the patient population received therapy. The most prescribed compounds, regardless of therapeutic schemes, were respectively Cycloferon (33.3%), Adefovir (26.9%), and Tenofovir (14.6%) (Figure 7). Monotherapy was prescribed in 75% (*n* = 520) of the cases treated, dual therapy in 18% (*n* = 127), and triple therapy in 7% (*n* = 46). The ranking of medicines used is presented in Figure 8 for monotherapies and in Tables 1 and 2 for combination therapies. The combination of Cycloferon with Adefovir was administered to almost 44% of patients receiving dual therapy, whereas Cycloferon and Lamivudine were used in 33% of the cases, and use of the other combinations ranged from 0.8% to 7.1%. Triple therapies relied essentially on the combination of Cycloferon, Lamivudine, and Adefovir (63%).

#### 3.3.2. Age, Gender, Origin, and Treatments

The median age of treated patients was 39 years ([IQR]: 30.6–47.8 years). Patients above median age were significantly more often treated than those under median (*p* < 0.0001). There was no difference between patients above or below the median age regarding compounds and therapeutic regimen employed. Treatments (globally) were independent of gender (*p* = 0.07), but treated men received more multitherapies than treated women (*p* = 0.02). There was no difference between therapeutic schemes according to the geographic origin of patients.

#### 3.3.3. VL and Treatment

There was a positive correlation between VL and number of administered medications (*p* = 0.0001). Combination therapies tended to be more often used as the VL increased.

84% (583/693) of treated patients had first detectable VL whereas a small subset of patients (16% of treated patients, *n* = 110/693) was treated despite the absence of detectable VL at first quantification.

In only 11.7% (81/693) of patients was the last VL undetectable; 65% of them were treated by monotherapy, 24% treated by dual therapy, and 11% treated by triple therapy.

#### 3.3.4. Aminotransferases and Treatments

Among patients receiving antiviral treatments, only 12% (*n* = 83/693) were reported to have a second follow-up determination of ALAT and 10.5% (73/693) for ASAT.

In these patients, mean values for ALAT and ASAT at initial sampling were significantly higher than those observed at the last follow-up sampling (86.6 versus 46.7 U/L, *p* = 0.01; 78.8 versus 45.3 U/L, *p* = 0.01, resp.).

## 4. Discussion

### 4.1. Cohort

Between January 2010 and November 2016, nearly 4,000 patients diagnosed in Lao public or private health care facilities came to CILM for HBV diagnosis confirmation and follow-up. In this cohort, patients came mainly from the Vientiane capital public health care system. This is certainly due to the central position of CILM in the capital, and also because public sector health care is less expensive than the private sector in Lao PDR. It is very unlikely that this figure reflects the prevalence of the disease, as large-scale HBV screening and treatment programs are almost nonexistent in Lao PDR. Patients come to diagnostic centres on their own discretion. The situation described in the present study is undoubtedly the tip of the iceberg: the true number of infected people in Lao PDR is probably much higher, but there is no previous study to confirm this hypothesis.

The cost of the serological diagnosis and quantification of HBV and some associated biomarkers have to be supported by people themselves, as most Lao patients do not benefit from health care insurance. This cost is around 67 US$, which represents a substantial expenditure for most people in the country. Considering that the GDP per capita in Laos was 1,500 US$ in 2016, the cost of such serological analysis constitutes almost 4% of the annual earnings for most Lao workers. Nevertheless, the number of patients attending CILM increased continually from around 200 patients in 2010 to more than 500 in 2016, with a peak of almost 800 patients in 2015 (Figure 1). This increase may reflect a dramatic increase of the number of new infections, as well as increased economic status, so people are more able to spend money on health care. This hypothesis is supported by the fact that the GDP per capita in Laos increased by 50% from 2009 to 2016, according to Trading Economics [9]. It is also possible that increasing public awareness about the dangers of HBV makes people more willing to be tested and treated.

In the present study, there were twice more men than women attending CILM for HBV diagnosis and follow-up, whatever their geographical origin. This is similar to most studies on HBV prevalence, in which generally a higher prevalence of HBV chronic carriers is found among males than females [10]; in the majority of literature it is generally accepted that HCC affects more men than women [11, 12]. In the Asia-Pacific region, the incidence of HCC for both genders has been shown to increase over the age of 40 [13]. However, in other developing regions like Peru, the occurrence of HCC in a younger patient population has been reported [14–16]. In the present study, the patient population had rather a classical age distribution, but it was striking to observe that patients under age 37 represented almost 50% of the individuals attending CILM (Figure 2). This observation is similar to those made by a consortium of researchers from various African countries (i.e., Ghana, Ivory Coast, Malawi, Nigeria, Sudan, Tanzania, and Uganda), which have recorded that about 40% of the individuals with HBV-related HCC in Africa develop their tumour before the age of 40 [17].

### 4.2. Viral Load

The presence of circulating viral genomes indicates an active chronic HBV infection, presaging chronic insult to the liver. Consequently, a high HBV VL has been identified among predictive factors of liver carcinogenesis. A survey performed in a prospective cohort of chronic HBV carriers in Taiwan clearly showed the significance of the association between serum HBV DNA levels and HCC risk [18]. Other biological parameters, such as aminotransferases, have been shown to be predictive as well, when associated with VL [18]. The rigorous follow-up of these serological parameters is then of utmost importance for the stratification of patients at risk.

HBV DNA quantification represents the best marker of viral replication [19]. In our patient population, VL ranged from <50 to 2.5 10^13^ IU/mL, the group of patients showing VL of 10^7^ being the most numerous.

There was no influence of the place of origin for the private/public status of health facility on the VL measured. Younger patients had a VL higher than older ones. Likewise, men displayed a higher VL than women. Almost 60% of the patient population had a VL higher than 10,000 IU/mL, the threshold value for high risk of developing HCC [20]. It is our opinion that this younger patient population should be closely monitored and treated accordingly.

Attention should also be focused on women over age 50, as a multicentre cross-sectional study in China established that the protective effect of female gender against the development of HBV-related cirrhosis gradually disappears after the age of 50 [21]. Furthermore, in the present study, 315 women of childbearing age (i.e., ≤37 years) had VL higher than 200,000 IU/mL. They should receive special attention as the use of antiviral therapies during the third trimester of pregnancy leads to a significant reduction in perinatal transmission [8].

### 4.3. Aminotransferases

In the present patient population, only 30% of patients attending CILM had aminotransferases records. Ideally, all patients with diagnosis of chronic HBV infection should have had aminotransferase monitoring. Unfortunately, the medical personnel transmitted incomplete laboratory-clinical charts most of the time, or patients themselves admitted their records had been lost.

In a survey conducted on 4,000 people in China, the upper cut-off values for ALAT and ASAT were, respectively, 22.15 IU/L and 25.35 IU/L in healthy men and 22.40 IU/L and 24.25 IU/L for healthy women [22]. In our study, median aminotransferase values at the first follow-up sampling were around 65 U/L for both enzymes. Mean ALAT and ASAT values in the most numerous median age groups (30–40 years) were almost three times higher than the normal level reported in the Chinese study. There was a significant difference between people under 37 and people above 37 for both biomarkers; younger people had higher levels of aminotransferase (30% higher for ALAT, 20% higher for ASAT).

According to Marcellin et al. [23], the risk of developing HCC in patients with compensated cirrhosis with normal ALAT levels is not low, and the long-term treatment is associated with reduced HCC risk, indicating that prompt treatment is necessary, even for those with normal aminotransferase levels.

When both serum HBV DNA and aminotransferase levels are elevated, treatment is recommended for patients with chronic hepatitis; however, for patients with compensated cirrhosis, treatment is recommended when serum HBV DNA levels are elevated, irrespective of serum aminotransferase levels [7]. In our cohort, 10.6% of patients had both a high level of HBV DNA (over 10,000 IU/mL) and aminotransferases, but among them only 2.4% received treatments. This should be seriously taken into account as it has been clearly demonstrated that HBV treatments, which suppress viral replication, can reverse liver fibrosis and cirrhosis and subsequently reduce the incidence of HCC [24, 25].

### 4.4. Treatments

Merely 18% of the patients reported receiving antiviral treatments and the number of people receiving treatment has been decreasing since 2012. Patients coming from Vientiane or the provinces received the same therapeutic attention. The more elevated the VL, the greater the number of drugs administered.

According to our data, eight different medicines were used by clinicians, irrespective of gender. Three therapeutic schemes were used: monotherapies, dual therapies, and triple therapies. The drug classes included nucleosides analogues including adenosine analogues (Adefovir and Tenofovir), guanosine analogues (Entecavir and Ribavirin), cytidine analogue (Lamivudine), immunostimulant (Cycloferon), and Interferon-like analogues (Interferon, Pegylated Interferon alpha-2a).

Monotherapy was reported for 75% of the treated group, dual therapy in 18%, and triple therapy in 7% of treated people; that is, less than 15%, 4%, and 2% of the whole population received monotherapy, dual therapy, or triple therapy, respectively.

Three drugs were predominantly prescribed: Cycloferon, Adefovir, and Tenofovir. Cycloferon and Adefovir were clearly decreasing since 2012, in favour of Tenofovir. Lamivudine and Pegasys had had a small increase in use between 2013 and 2014. Tenofovir was the only drug that persisted at a stable and high level from 2013 to 2016. Monotherapy was the primary treatment scheme of choice in Laos over the last 7 years.

Among the monotherapies, nucleosides analogues were preferred as they represented 62.3% of the treatments.

Adefovir and Tenofovir which are both acyclic nucleoside phosphonate analogues, reverse-transcriptase inhibitors (a crucial HBV enzyme [26]), were used for almost 27% and 21% of treated patients, respectively. Adefovir is almost three times less expensive than Tenofovir (see below).

Entecavir and Tenofovir monotherapy has been shown to achieve inhibition of HBV replication in almost all adherent patients [27]. These drugs are known to improve liver fibrosis and reverse cirrhosis in a majority of patients. In Laos, Tenofovir (21%) was preferred over Entecavir, which was anecdotally used (i.e., 0.8% of monotherapies).

Although Ribavirin is part of the World Health Organization's list of essential medicines, the most important medication needed in a basic health system [28], it was rarely used (0.8% of monotherapies).

Finally, Lamivudine was mentioned in only 12.7% of monotherapies, although it is the least expensive. It was probably little used because of its tendency to cause resistance in most patients over a five-year period [29].

Interferon-like drugs and immunostimulants were used in 37.7% of monotherapies. Pegylated Interferon-*α* (around 5.4%) and Interferon (1.9%) are cytokines that are claimed to have a limited duration of therapy in HBV therapeutic schemes (if pursued for at least 48 weeks), the absence of drug resistance, and an opportunity to obtain a durable posttreatment response [27]. Cycloferon is an early inductor of types 1 and 2 Interferon, administered orally or through the parenteral route. This is an association between acridone acetic acid and meglumine. Imported from Russia into Laos, it is particularly cheap and was used extensively when Russian doctors were present in the public health system. Cycloferon was used in 30.4% of treated people in our survey mostly alone and sometimes in combination. According to these doctors (some are now working in private clinics in Vientiane), Cycloferon is particularly effective but should be administered in combination with other antiviral drugs. Nevertheless, only 25% of treated patients received multitherapies. Interestingly, more than 90% of the combinations associate nucleoside analogues and Interferon-like molecules, especially Cycloferon.

Unfortunately, medical staff did not indicate which criteria were used to select a therapeutic scheme preferentially.

Strikingly, 11% of patients had both a high level of VL (≥10,000 IU/mL) and high liver enzymes (>40 U/L) but among them only 2% received treatments. Moreover, among all treated patients, only 12% had undetectable VL after treatment. Nevertheless, results on the impact of therapies on VL and aminotransferases should be cautiously interpreted, as we cannot be confident that the patients strictly respected their treatment protocols.

To reduce the length and the associated cost of the therapy, some authors [30] suggested discontinuing long-term nucleoside therapy, if close follow-up can be ensured in patients without advanced liver disease. Therefore, precise valuation of disease status and appropriate beginning of antiviral therapy should be clearly redesigned for the cost-effective management of patients in Lao PDR.

### 4.5. Other Parameters

Additional systematic biological parameters should be introduced to improve HBV infection follow-up in Laos. As mentioned above, determination of HBeAg and anti-HBe should be implemented to differentiate patients in the immune clearance phase from those in the reactivation phase that usually heralds severe complications such as HCC. Platelet and aminotransferase levels should also be taken into account to estimate an index to detect subjects with severe fibrosis when >3.25 [31].

AFP reinforced by transabdominal ultrasonography has also been shown to be extremely valuable parameter giving key information on the ongoing tumour process and should be then added in the clinical-biological monitoring of patients [30].

Finally, other variables known to be associated with HCC risk (e.g., socioeconomic status, alcohol intake, smoking, excess weight, infection with* Opisthorchis*, and drug treatment) should be collected in the near future to delineate the degree of HCC risk in Laos more accurately.

## 5. Conclusion

The picture drawn from the results obtained from this survey is alarming. More than 60% of the patients included had a very high HBV VL; they were mostly young people with aggravating factors such as high aminotransferases. It is also evident from our survey that the level of treatment is critically low. It is obvious that all of the factors are already in place for an increase in HCC cases in the near future.

In Lao PDR, tools and skills are present to rewrite the scenario of this predictable health catastrophe. Only an immediate political decision could slow this fatal trend by firstly improving large-scale national vaccination campaigns, which have been shown to be very effective in Asia [21], secondly, supporting a very precise and vigorous serial biological and medical imaging follow-up of patients, and, finally, putting a system in place for management of at-risk infected patients.

This strategy could be successful only if supplemented by efficient prevention campaigns, better coordination between medical staff and the laboratory, improvement of up-to-date online surveillance networks, and, last but not least, affordability of diagnosis and treatments.

## Figures and Tables

**Table 1:** Type of drugs, number, and % on total and dual treatments.

Dual therapies	Nb	% of total treatment (*n* = 693)	% of dual therapy (*n* = 127)
Cycloferon + Adefovir	55	7.9%	43.3%
Cycloferon + Lamivudine	42	6.1%	33.0%
Adefovir + Lamivudine	9	1.3%	7.1%
Cycloferon + Tenofovir	5	0.7%	4.0%
Tenofovir + Adefovir	4	0.6%	3.2%
Tenofovir + Lamivudine	4	0.6%	3.2%
Pegasys + Tenofovir	3	0.4%	2.4%
Pegasys + Adefovir	2	0.3%	1.6%
Entecavir + Tenofovir	1	0.1%	0.8%
Interferon + Cycloferon	1	0.1%	0.8%
Entecavir + Pegasys	1	0.1%	0.8%

*Total*	*126*	*18.2%*	

**Table 2:** Type of drugs, number, and % on total and triple treatments.

Triple therapies	Nb	% of total treatment	% of triple therapy
Cycloferon + Lamivudine + Adefovir	29	4.2%	63.0%
Cycloferon + Lamivudine + Pegasys	3	0.4%	6.5%
Adefovir + Lamivudine + Tenofovir	2	0.3%	4.3%
Adefovir + Tenofovir + Cycloferon	2	0.3%	4.3%
Lamivudine + Cycloferon + Ribavirin	2	0.3%	4.3%
Adefovir + Cycloferon + Entecavir	1	0.1%	2.2%
Adefovir + Cycloferon + Interferon	1	0.1%	2.2%
Adefovir + Cycloferon + Pegasys	1	0.1%	2.2%
Cycloferon + Interferon + Entecavir	1	0.1%	2.2%
Cycloferon + Lamivudine + Entecavir	1	0.1%	2.2%
Cycloferon + Lamivudine + Tenofovir	1	0.1%	2.2%
Interferon + Cycloferon + Lamivudine	1	0.1%	2.2%
Pegasys + Ribavirin + Tenofovir	1	0.1%	2.2%

*Total*	*46*	*6.6%*	
